# Cyanoacrylate Injection Compared with Band Ligation for Acute Gastric Variceal Hemorrhage: A Meta-Analysis of Randomized Controlled Trials and Observational Studies

**DOI:** 10.1155/2014/806586

**Published:** 2014-04-24

**Authors:** Xiaohua Ye, Jiaping Huai, Yanping Chen

**Affiliations:** ^1^Department of Gastroenterology and Hepatology, Jinhua Municipal Central Hospital, Jinhua Hospital of Zhejiang University, Jinhua, Zhejiang 321000, China; ^2^Department of Critical Care Medicine, Jinhua Municipal Central Hospital, Jinhua Hospital of Zhejiang University, Jinhua, Zhejiang 321000, China

## Abstract

*Background*. Cyanoacrylate injection (GVO) and band ligation (GVL) are effective treatments for gastric variceal hemorrhage. However, data on the optimal treatment are still controversial. *Methods*. For our overall analysis, relevant studies were identified from several databases. For each outcome, data were pooled using a fixed-effect or random-effects model according to the result of a heterogeneity test. *Results*. Seven studies were included. Compared with GVL, GVO was associated with increased likelihood of hemostasis of active bleeding (odds ratio [OR] = 2.32; 95% confidence interval [CI] = 1.19–4.51) and a longer gastric variceal rebleeding-free period (hazard ratio = 0.37; 95% CI = 0.24–0.56). No significant differences were observed between GVL and GVO for mortality (hazard ratio = 0.66; 95% CI = 0.43–1.02), likelihood of variceal obliteration (OR = 0.89; 95% CI = 0.52–1.54), number of treatment sessions required for complete variceal eradication (weighted mean difference = −0.45; 95% CI = −1.14–0.23), or complications (OR = 1.02; 95% CI = 0.48–2.19). *Conclusion*. GVO may be superior to GVL for achieving hemostasis and preventing recurrence of gastric variceal rebleeding but has no advantage over GVL for mortality and complications. Additional studies are warranted to enable definitive conclusions.

## 1. Introduction

Gastroesophageal variceal hemorrhage is a severe complication of chronic liver disease [1]. Although patient outcomes for variceal hemorrhage have improved over the past few decades, it is still a major cause of death in patients with portal hypertension; indeed gastric variceal hemorrhage (GVH) occurs in up to 20% of such patients [2]. Although GVH occurs less often than esophageal variceal hemorrhage (EVH), it has a worse prognosis, more severe blood loss, a higher rate of rebleeding, and a higher mortality [2–4]. A variety of methods have been employed for management of GVH [5, 6]. These include traditional methods such as vasoactive agents (somatostatin, terlipressin, or octreotide) and balloon tamponades, endoscopic therapies such as endoscopic injection sclerotherapy, thrombin injection, band ligation, and endoscopic obturation using tissue adhesives (glue), radiologic interventions such as transjugular intrahepatic portosystemic stent shunts and balloon-occluded retrograde transvenous obliteration, and surgical interventions. However, data to support the efficacy of some of these methods are scarce. Transjugular intrahepatic portosystemic stent shunt is effective for controlling active gastric variceal (GV) bleeding [7] and is considered a salvage therapy for patients who fail endoscopic treatment [5, 6]. However, no optimal treatment for GVH has been firmly established.

Endoscopic modalities including endoscopic injection of N-butyl-2-cyanoacrylate (GVO) and band ligation (GVL) have been successful for treating GVH in many clinical centers owing to the availability and relative effectiveness of these methods [8, 9]. The results of nonrandomized trials indicate that GVO has a higher success rate for controlling GVH compared with use of other sclerosants [10, 11]; however, optimal management of GVH remains unclear because of a lack of information from a large randomized controlled trial (RCT). Several RCTs have compared the efficacy of GVO and GVL for treatment of GVH, but these studies have yielded conflicting results [8, 9, 12, 13]. Lo et al. [8] reported that GVO was more effective than GVL for managing GVH, whereas other RCTs showed no significant differences [9, 12, 13]. Furthermore, there are conflicting opinions regarding the best management of gastroesophageal varices type 1 (GOV1) [5]. Some endoscopists suggest that GVH from GOV1 should be treated in the same manner as EVH [14]. However, only one RCT has specifically addressed the comparison of GVO and GVL for controlling GVH from GOV1 [12]. Considering that GVH is a potentially fatal complication with limited therapeutic options, it is crucial to understand the efficacy and safety of GVO versus GVL for managing GVH. We therefore performed a meta-analysis to incorporate the most recent data from clinical trials and provide a precise estimation of the clinical benefits and risks of GVO and GVL for the treatment of GVH.

## 2. Materials and Methods

### 2.1. Identification of Relevant GVH Studies

Studies were identified by searching the databases of MEDLINE, EMBASE, the Cochrane Library, and Google Scholar for trials concerning GVH occurring January 1990 to January 2014.The following search terms were used: gastric varices (or gastric varic*), cirrhosis (or cirrho*, liver-cirrhosis, portal hypertension*, and hypertension-portal), band ligation (or ligat*, ligation, and banding ligation), and cyanoacrylate (or N-butyl-2-cyanoacrylate). The search was limited to human studies and reports of clinical trials. The detailed search strategy is shown in Table 5. To maximize search efficiency, we planned the search strategy with a professional librarian. The reference lists of pertinent articles were also manually reviewed to identify additional studies.

### 2.2. Study Selection and Data Extraction

Two reviewers independently evaluated the identified studies according to prespecified selection criteria: (1) inclusion of patients with cirrhosis based on results of clinical, laboratory, and imaging studies; (2) inclusion of patients with clinical signs of hematemesis, coffee ground vomitus, hematochezia, or melena; (3) inclusion of patients with endoscopic signs of an active spurting or oozing from gastric varices; (4) inclusion of patients with adherent blood clots, white nipple signs, or erosions on gastric varices; (5) consideration of at least one of the following outcomes—cessation of active bleeding, gastroesophageal variceal rebleeding, mortality, variceal obliteration, and complications; and (6) comparison of GVO versus GVL. The following exclusion criteria were also set: (1) study did not concern human subjects; (2) data were not meta-analyzable (i.e., letter, review, practice guideline, editorial, case report, consensus statement, etc.) or (3) duplicated reports. For studies in which insufficient data were available to assess eligibility, we attempted to contact the author to obtain the original data. Differences between the two reviewers were resolved by consensus discussion. In total, 7 studies were included in our meta-analysis. Descriptive information about the subjects, study design, interventions, clinical outcomes, and features of the analysis was extracted from eligible studies using a standardized data abstraction form.

### 2.3. Assessment of Study Quality

For each RCT, potential bias was assessed independently by two reviewers using the Cochrane risk of bias tool [18]. The risk of bias was assessed based on the following domains: sequence generation, allocation concealment, blinding of participants, personnel and outcome assessors, incomplete outcome data, selective reporting, and other bias [18]. Each of these domains was rated as “high risk,” “low risk,” or “unclear.” The quality of nonrandomized studies was assessed using the Newcastle-Ottawa scale with some modifications to match the needs of our study [19, 20]. The criteria included three categories: (1) patient selection (three items); (2) comparability of the two study arms (two items); and (3) assessment of outcome (two items). Studies were awarded a maximum of one star per item in the patient selection and assessment of outcome categories and a maximum of two stars per item in the comparability of the two study arms category. Studies were graded on an ordinal star scoring scale. Score could range from 0 to 9, with higher scores indicating studies of higher quality. Studies achieving six or more stars were considered to be of high quality. Quality of studies was assessed independently by two reviewers. Discrepancies in the evaluation of quality were resolved through discussion between the reviewers.

### 2.4. Statistical Analysis

Stata software, version 12.0 (Stata Corporation, College Station, TX), was used for all data analyses. The outcome measures were odds ratio (OR) for dichotomous data and weighted mean difference for continuous data, and both are reported with 95% confidence interval (CI). Cumulative GV rebleeding-free survival and overall survival were evaluated by pooled Cox proportional hazard ratios (HRs) with corresponding 95% CI using a calculation sheet as described [21]. Briefly, all the reported summary statistics were entered, and the spreadsheet then calculated the results by all possible methods. Results from all methods were provided in a single output screen, which facilitated comparison. HR and 95% CI were estimated from studies that presented *P* values and the total number of events and patients in each group [8, 9, 17] and from the one study that reported the time interval, mortality data, and the number of patients at risk [13]. A fixed effect or random effects model was used to pool the data according to the result of a statistical heterogeneity test [22]. Heterogeneity between studies was evaluated using *Q*-statistic and *I* ^2^ tests [23]. Publication bias was evaluated using Begg's funnel plot and Egger's test [24, 25]. Subgroup analyses were performed according to prespecified criteria including study design (RCT or non-RCT), GV type, and proportion of patients with hepatocellular carcinoma (HCC). For all analyses, a *P* value of less than 0.05 was considered to reflect statistical significance.

## 3. Results

### 3.1. Search Results and Characteristics of Individual Studies

**Figure 1:**
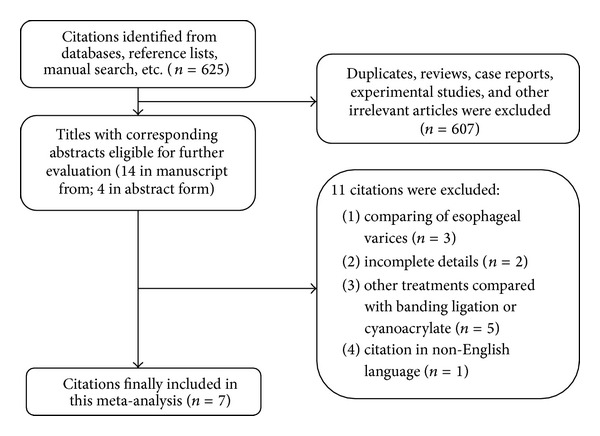
Flow chart of the study selection.

The initial search yielded 625 citations, and 607 were excluded by inspection of the titles or abstracts because they were duplicates, reviews, experimental studies, or irrelevant to our analysis. Eleven additional studies were excluded because they had comparisons of esophageal varices (*n* = 3), incomplete outcomes (*n* = 2), inadequate intervention methods (*n* = 5), or were published in a non-English language (*n* = 1). As a result, seven studies [8, 9, 12, 13, 15–17] were included in the meta-analysis (Figure 1).

The seven studies included 648 patients, and the sample size varied from 37 to 162 across the studies. We contacted the investigators of two included studies [12, 13] with requests for additional data, and the investigators of one study [13] indeed provided additional data.  Table 1 lists the clinical characteristics of the patients. The percentage of the study sample that was male ranged from 56.8% to 86.9%. Two studies [8, 9] (157 patients) included all types of gastric varices according to Sarin classification [4], three studies [12, 15, 16] (396 patients) included only patients with GOV1, and two studies [13, 17] (85 patients) included patients with GOV1 and GOV2.

**Figure 2:**
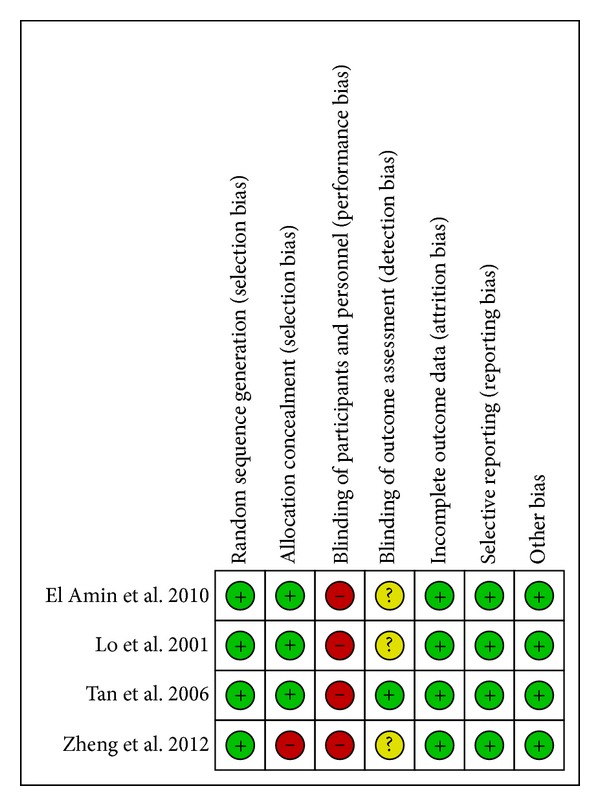
Risk of bias assessment for RCTs. Reviewers' judgment of the risk of bias for each item for each of the four RCTs included in the meta-analysis. Green-colored symbol corresponds to low risk of bias, yellow corresponds to unclear risk of bias, and red corresponds to high risk of bias.

The risk of bias in each of the four RCTs is shown in Figure 2 [8, 9, 12, 13]. Random sequence generation and allocation sequence concealment were classified as “low risk” in four [8, 9, 12, 13] and three [8, 9, 12] trials, respectively. Blinding of participants or personnel was not conducted in any of the four RCTs owing to infeasibility of study design. Blinding of outcome assessment was specifically reported in one trial [9]. The number of and reason for withdrawals/dropouts were reported in detail in all RCTs. None of the included trials had selective outcome reporting. There was no potential source of other bias detected in the included RCTs.

Table 2 shows the methodological quality of the three nonrandomized studies [15–17]. There was one prospective study [17] and two retrospective studies [15, 16], and all three were of high quality (Newcastle-Ottawa scale score ≥ 6).

### 3.2. Hemostasis of Active Bleeding and GV Rebleeding

**Figure 3:**
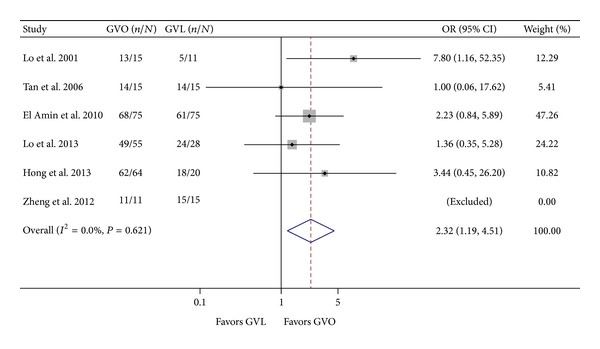
Comparison of hemostasis of active bleeding in the GVO and GVL groups. GVO, cyanoacrylate injection; GVL, band ligation; CI, confidence interval; OR, odds ratio.

Six studies [8, 9, 12, 13, 15, 16] (611 patients) compared the effectiveness of GVO and GVL in achieving hemostasis of active bleeding. One RCT [8] demonstrated GVO to be more advantageous than GVL, and the remaining studies [9, 12, 13, 15, 16] showed no significant difference between GVO and GVL. In the analysis of pooled studies, a fixed effect model indicated that hemostasis for active bleeding was more likely in the GVO group than in the GVL group (OR = 2.32; 95% CI = 1.19–4.51; Figure 3). There was no significant heterogeneity across studies (*I* ^2^ = 0.0%; *P* = 0.621).

**Figure 4:**
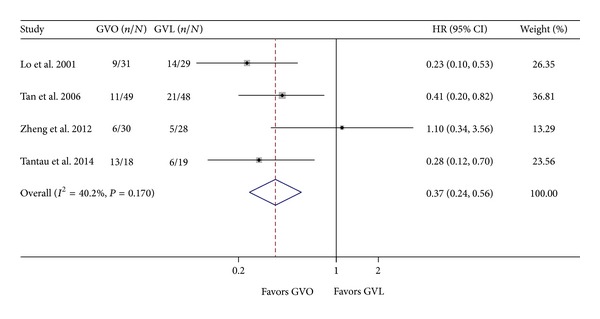
Comparison of rebleeding of gastric varices in the GVO and GVL groups. GVO, cyanoacrylate injection; GVL, band ligation; CI, confidence interval; HR, hazard ratio.

Data on GV rebleeding were extracted from four studies [8, 9, 13, 17]. In the pooled data analysis, a fixed effect model indicated that GVO was associated with a statistically significant 63% reduction in the hazard of GV rebleeding (HR = 0.37; 95% CI = 0.24–0.56; Figure 4). There was no significant heterogeneity across studies (*I* ^2^ = 40.2%; *P* = 0.17).

### 3.3. Mortality

**Figure 5:**
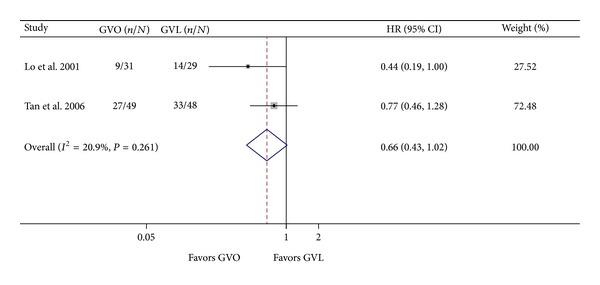
Comparison of mortality in the GVO and GVL groups. GVO, cyanoacrylate injection; GVL, band ligation; CI, confidence interval; OR, hazard ratio.

Two studies reported cumulative overall patient mortality [8, 9]. GVO was associated with a nonsignificant reduction in mortality (HR = 0.66; 95% CI = 0.43–1.02). There was no significant heterogeneity between the two studies (*I* ^2^ = 20.9%; *P* = 0.261; Figure 5).

### 3.4. Variceal Obliteration and Treatment Sessions

**Figure 6:**
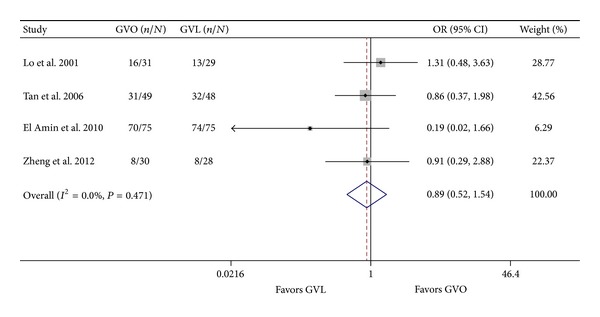
Comparison of variceal obliteration in the GVO and GVL groups. GVO, cyanoacrylate injection; GVL, band ligation; CI, confidence interval; OR, odds ratio.

Four RCTs (365 patients) compared the efficacy of GVO and GVL with respect to variceal obliteration [8, 9, 12, 13]. We noticed a trend that variceal obliteration was more common in the GVO arm in one RCT [8] but more common in the GVL arm in the other three RCTs [9, 12, 13]; however, none of the differences were statistically significant. In the pooled data analysis, a fixed effect model indicated no significant difference between the GVO and GVL groups (OR = 0.89; 95% CI = 0.52–1.54; Figure 6). There was no significant heterogeneity across studies (*I* ^2^ = 0.0%; *P* = 0.471).

**Figure 7:**
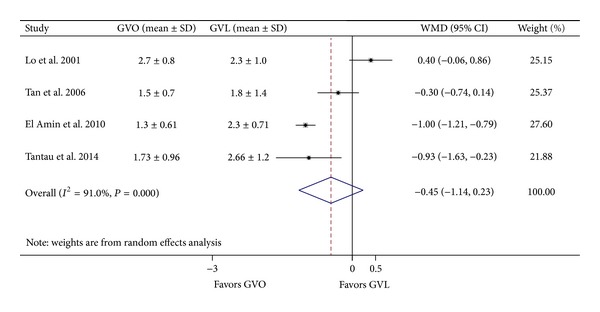
Comparison of number of treatment sessions in the GVO and GVL groups. GVO, cyanoacrylate injection; GVL, band ligation; CI, confidence interval; WMD, weighted mean difference.

Four studies [8, 9, 12, 17] reported the number of treatment sessions required to achieve complete variceal eradication. In the pooled analysis, a random effects model indicated no significant difference between the GVO and GVL groups (weighted mean difference = –0.45; 95% CI = −1.14–0.23; Figure 7). However, there was significant intertrial heterogeneity (*I* ^2^ = 91.0%; *P* < 0.001).

### 3.5. Complications

**Figure 8:**
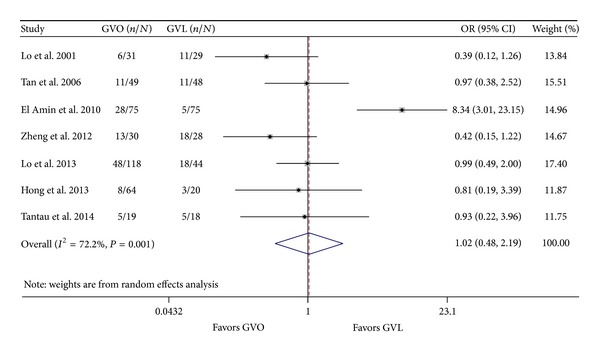
Comparison of the overall incidence of complications in the GVO and GVL groups. GVO, cyanoacrylate injection; GVL, band ligation; CI, confidence interval; OR, odds ratio.

All seven trials (648 patients) reported the occurrence of complications in the GVO and GVL groups [8, 9, 12, 13, 15–17]. Overall, complications occurred in 119 patients (39.02%) of the GVO group and 71 patients (27.10%) of the GVL group. In six studies there were fewer complications in the GVO group than in the GVL group, but the difference in each case was not significant [8, 9, 13, 15–17]; in the other study there were significantly fewer complications in the GVL group than in the GVO group [12]. In the pooled data analysis, the incidence of complications was similar in the GVO and GVL groups (OR = 1.02; 95% CI = 0.48–2.19; Figure 8). However, there was significant intertrial heterogeneity (*I* ^2^ = 72.2%; *P* = 0.001). We considered that the source of intertrial heterogeneity might be ascribed to the study by El Amin et al. [12], as the definition of complication was not strictly limited in this study yet conditions like hepatic encephalopathy and hepatorenal syndrome were included. It is likely that these conditions were not caused by GVO or GVL* per se* but rather were associated with cirrhosis. When we excluded this study, the intertrial heterogeneity became nonsignificant (OR = 0.75; 95% CI = 0.49–1.14; *I* ^2^ = 0.0%, *P* = 0.657). The most common complications related to GVO and GVL were ulcers or ulcer bleeding and infections. Infections included sepsis, bacteremia, pneumonia, spontaneous bacterial peritonitis, urinary tract infection, fever, and leukocytosis. Data on specific categories of complications were reported in all studies, and the summary results are shown in Table 3. The incidence of ulcers or ulcer bleeding was significantly lower in the GVO group than in the GVL group (OR = 0.32, 95% CI = 0.17–0.67; *I* ^2^ = 17.7%, *P* = 0.302).

### 3.6. Subgroup Analysis

Table 4 presents the results of the subgroup analyses. Analyses using only the data from RCTs indicated that the results were consistent with the full analysis between the GVO and GVL groups with respect to hemostasis of active bleeding (OR = 2.64, 95% CI = 1.15–6.05; *I* ^2^ = 0.0%, *P* = 0.407), GV rebleeding (OR = 0.43, 95% CI = 0.20–0.92; *I* ^2^ = 56.0%, *P* = 0.103), number of treatment sessions (weighted mean difference = −0.46; 95% CI = −1.58–0.66; *I* ^2^ = 95.3%, *P* < 0.001), and complications (OR = 0.58; 95% CI = 0.32–1.06; *I* ^2^ = 0.0%, *P* = 0.386).

Because HCC is an important risk factor for the prognosis of patients with GVH [9, 16], we performed a subgroup analysis based on the proportion of patients with HCC. Hemostasis of active bleeding was not statistically different between the GVO and GVL groups in studies with a low proportion (< median, 19.95%) of patients with HCC [12, 15]. Moreover, the GVO group had fewer number of treatment sessions than the GVL group in studies with a low proportion of patients with HCC (weighted mean difference = −0.99; 95% CI = −1.20, −0.79) [12, 17].

Subgroup analyses were also performed for nonrandomized studies and different GV types. Among outcomes that were investigated in more than two studies, GVO was associated with an increased likelihood of hemostasis of active bleeding (OR = 2.34; 95% CI = 1.17–4.69; *I* ^2^ = 0.0%, *P* = 0.456) [8, 12, 15, 16] and a decreased risk of ulcers after hemostasis or ulcer bleeding (OR = 0.37; 95% CI = 0.18–0.76; *I* ^2^ = 51.2%, *P* = 0.129) [12, 15, 16] in patients with GOV1. However, hemostasis of active bleeding did not differ significantly between GVO and GVL in two retrospective studies (OR = 1.81; 95% CI = 0.59–5.60; *I* ^2^ = 0.0%, *P* = 0.456) [15, 16]. There was no significant difference in hazard of GV rebleeding between the GVO and GVL groups in studies that only included patients with GOV1 or GOV2 (HR = 0.52; 95% CI = 0.14–1.99; *I* ^2^ = 70.0%, *P* = 0.068) [13, 17].

### 3.7. Publication Bias

**Figure 9:**
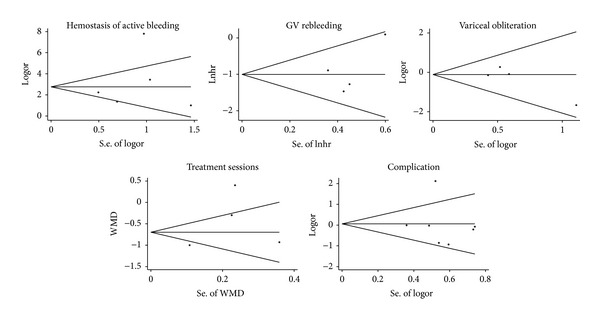
Begg's funnel plot with pseudo 95% confidence limits showing the symmetrical distribution of the included studies.

Publication bias was assessed for all outcomes (Figure 9). There was no evidence of publication bias as demonstrated by the Egger's test or Begg's test (*P*_Begg_ = 0.624, *P*_Egger_ = 0.593 for hemostasis of active bleeding; *P*_Begg_ = 0.497, *P*_Egger_ = 0.429 for GV rebleeding; *P*_Begg_ = 0.497, *P*_Egger_ = 0.221 for variceal obliteration; *P*_Begg_ = 0.497, *P*_Egger_ = 0.390 for treatment sessions; *P*_Begg_ = 0.881, *P*_Egger_ = 0.810 for complications).

## 4. Discussion

Although GVH is less common than EVH, it results in more blood loss and higher mortality and thus represents a more challenging clinical problem than EVH [2–4]. Unfortunately, data on the optimal management for GVH is limited. Several endoscopic treatment modalities for GVH, including injection of sclerosants or thrombin and GVL, have been applied, but such strategies for GVH are less well established than those for EVH [26–29].

Previous studies have reported the use of injection sclerotherapy for treating GVH [3, 30]. However, injection sclerotherapy is usually associated with a high rebleeding rate and a frequent requirement for surgical intervention and thus is regarded as only a temporary hemostatic modality [3, 30]. GVO using N-butyl-2-cyanoacrylate may have a higher success rate than sclerotherapy for controlling GVH [10, 11]. The superiority of band ligation for EVH is well documented with regard to both efficacy and safety, whereas the efficacy and safety of band ligation for GVH is uncertain [29, 31]. GVO is the most effective therapy for GOV2 bleeding, whereas either GVO or GVL can be applied for GOV1 bleeding [16]. Currently, among the endoscopic therapeutic techniques, GVO is recommended as a first-line endoscopic therapy because it is supported by the strongest evidence [32–34]. However, data on GVO versus GVLremains somewhat conflicting.

 The foremost indication for endoscopic treatment in patients with GVH is cessation of active bleeding. One trial with a small number of cases showed a better benefit from GVO than GVL [8] for arresting active bleeding, whereas the other studies included in our meta-analysis reported equal efficacy of GVO and GVL. The meta-analysis showed that GVO was associated with an increased likelihood of arresting active bleeding in GVH. By contrast, previous studies have suggested that both GVO and GVL can be effective at arresting bleeding in patients with GOV1 [35, 36]. This is mainly because GOV1 extends beyond the gastroesophageal junction and is always associated with esophageal varices [35, 37]. GVL seems to be more effective at arresting GOV1 bleeding than GVO, and thus we extracted data on patients with GOV1 [8, 12, 15, 16]. Synthesis of these results showed an increased likelihood of arresting active bleeding with GVO than with GVL. There was no significant heterogeneity across these studies, supporting the robustness of combining the data; this implies that GVO may provide an increased likelihood of arresting active bleeding in GOV1.

Our meta-analysis showed that the rate of GV rebleeding was lower with GVO than with GVL. Three trials showed a lower hazard of GV rebleeding with GVO [8, 9, 17], whereas one study did not find a statistically significant difference between GVO and GVL [13]. These conflicting results may be attributable to heterogeneity between these studies because different proportion of patients had HCC and acute treatment basis (versus elective basis), and there were various other differences in technical applications. A possible explanation may be that GVO could obliterate collaterals over a wider area and in deeper layers than GVL, whereas GVL's effect is limited on only the superficial collaterals in the mucosal and submucosal layers [9].

Complete eradication of varices was evaluated in five studies [8, 9, 12, 13] that were included in our meta-analysis. The pooled data analysis did not detect any difference in complete eradication of varices between the GVO and GVL groups. The number of treatment sessions required to achieve variceal obliteration did not differ significantly between the GVO and GVL groups in the combined data analysis, and there was remarkable intertrial heterogeneity. Subgroup analysis failed to identify the source of heterogeneity, but it may be ascribed to the different techniques or methods applied in different trials (single versus multiple ligators, dose of cyanoacrylate, number of rubber bands, etc.).

Evaluation of an endoscopic therapeutic modality requires not only determining its efficacy but also assessing its potential side effects. The overall incidence of complications did not differ between the GVO and GVL groups. However, there was remarkable intertrial heterogeneity. This may be partly explained by broader inclusion criteria for complications in the study by El Amin et al. [12]. In addition, the etiology of cirrhosis in this study differed from that in other studies because a significant number of patients presented with schistosomiasis. Ulcer bleeding after hemostasis is a common local complication. Our meta-analysis showed that ulcer bleeding after hemostasis occurred more frequently with GVL than with GVO in all patients and in the subgroup of GOV1 patients. Other complications such as infection, vascular events (cerebral vascular accident or embolism), pain, dysphagia, hepatic encephalopathy, and hepatorenal syndrome did not differ between the two treatment groups, and heterogeneity was not significant in any category.

Although no publication bias was observed, our study has potential limitations. First, although extensive literature and abstract review was performed to minimize bias in the results, some studies were observational in design and thus the assignment of patients to different interventions was subject to selection bias. Low quality studies can result in a distortion of the summary effect estimate. Subgroup analysis according to study design was performed to address this issue, and the results indicated that most outcomes were consistent across studies of different design. Second, we used the Newcastle-Ottawa scale to assess the quality of nonrandomized studies. This scale has been used extensively in other studies, but some limitations have been reported by Stang [38]. Third, only limited information could be obtained from the abstracts that were included in our study. Therefore, we contacted the author of the abstract and part of the data was obtained. Finally, heterogeneity existed in some pooled estimates; thus, these results must be interpreted with caution.

In summary, to the best of our knowledge, this is the first study to systematically review the literature on optimal endoscopic management of GVH. GVO may be superior to GVL for arresting active bleeding and reducing the risk of rebleeding. No evidence was found that GVO reduced mortality, complications, or the number of sessions required for variceal obliteration or improved variceal obliteration.

## Figures and Tables

**Table 1:** Characteristics of included studies.

Author	Year	Region	Publication type	Design	Sample size^1^	Mean age (years)	Gender (M/F)	Child-Pugh class^2^	GV type^3^	HCC^1^	Mean followup^1^ (days)
Lo et al. [8]	2001	Taiwan single center	Article	RCT	31/29	56.6	46/14	13/33/14	41/14/5	7/10	420/270
Tan et al. [9]	2006	Taiwan single center	Article	RCT	49/48	61.6	69/28	25/51/21	53/25/19	23/23	610.58 ± 603.04/680.67 ± 710.54
El Amin et al. [12]	2010	Egypt multicenter	Article	RCT	75/75	51.0	108/42	35/72/43	150/0/0	0/0	180/180
Zheng et al.^6^ [13]	2012	China single center	Abstract	RCT	30/28	NR	48/10	25/26/7	25/13/0	NR	≥365^5^
Lo et al. [15]	2013	Taiwan single center	Article	Retro	118/44	50.6	140/22	36/55/61	162/0/0	20/8	42/42
Hong et al. [16]	2013	Korea single center	Article	Retro	73/11	57.0^4^	73/11	45/33/6	84/0/0	16/3	NR
Tantau et al. [17]	2014	Romania single center	Article	Pros	19/18	61.2	21/16	11/18/8	22/15/0	1/1	427.26 ± 214.16/406.21 ± 213.23

^1^Data are for patients receiving GVO/GVL; ^2^data are for Child-Pugh class A/B/C; ^3^data are for GOV1/2/IGV; ^4^value is the median; ^5^reported as “at least 1-year followup”; ^6^data from this study were obtained directly from the author.

RCT: randomized controlled trial; Retro: retrospective; Pros: prospective; GOV: gastroesophageal varices; IGV: isolated gastric varices; HCC: hepatocellular carcinoma; NR: not reported.

**Table 2:** Methodological quality of nonrandomized studies.

Author	Selection			Comparability		Assessment of outcome		NOS score
	Item 1	Item 2	Item 3	Item 4	Item 5	Item 6	Item 7	
Lo et al. [15] 2013	∗	∗	∗		∗∗	∗	∗	7
Hong et al. [16] 2013	∗	∗	∗	∗	∗	∗	∗	7
Tantau et al. [17] 2014	∗	∗	∗	∗∗	∗∗	∗	∗	9

Item 1: inclusion criteria reported; item 2: generalizability of patients with cyanoacrylate injection to population with acute gastric variceal hemorrhage; item 3: generalizability of patients with banding ligation to population with acute gastric variceal hemorrhage; item 4: age, gender, and etiology (2 stars if yes to all; 1 star if one of these parameters was not reported; no stars if the two groups differed); item 5: presence of hepatocellular carcinoma, Child-Pugh class, encephalopathy (2 stars if yes to all; 1 star if one of these parameters was not reported; no stars if the two groups differed); item 6: clearly defined outcome of interest; item 7: adequacy of followup (1 star if followup >90%). NOS: Newcastle-Ottawa scale.

**Table 3:** Comparison of complications between GVO and GVL.

Variable	Number of patients	Combined OR (95% CI)	Model	*I* ^2^ (%)	*P* value for heterogeneity
Ulcers/ulcer bleeding	5 (493)	0.32 (0.17–0.67)	Fixed effect	17.7	0.302
Infections	7 (648)	0.94 (0.58–1.50)	Fixed effect	0.0	0.601
Vascular events^1^	4 (403)	1.76 (0.35–8.85)	Fixed effect	0.0	0.941
Pain	2 (220)	0.54 (0.19–1.54)	Fixed effect	42.9	0.186
Dysphagia	1 (150)	5.29 (0.60–46.38)	—	—	—
HE	1 (150)	3.08 (0.31–30.34)	—	—	—
HRS	1 (150)	4.17 (0.45–38.21)	—	—	—

GVO: cyanoacrylate injection; GVL: band ligation; HE: hepatic encephalopathy; HRS: hepatorenal syndrome; CI: confidence interval; OR: odds ratio.

^1^Vascular events include cerebral vascular accident and embolism.

**Table 4:** Results of subgroup analyses.

Variable	Number of patients	Combined results (95% CI)	Model	*I* ^2^ (%)	*P* value for heterogeneity
Hemostasis of active bleeding					
Study design					
RCT	4 (232)	2.64 (1.15, 6.05)	Fixed effect	0.0	0.407
Retrospective	2 (167)	**1.81 (0.59, 5.60)**	Fixed effect	0.0	0.456
GV type					
Overall	1 (30)	1.00 (0.06, 17.62)	—	—	—
GOV1+2	1 (26)	7.80 (1.16, 52.35)	—	—	—
GOV1	4 (337)	2.34 (1.17, 4.69)	Fixed effect	0.0	0.599
Proportion of HCC					
Higher (>median)	3 (241)	3.87 (1.11, 13.52)	Fixed effect	0.0	0.500
Lower (<median)	2 (312)	**1.89 (0.86, 4.51)**	Fixed effect	0.0	0.562

GV rebleeding					
Study design					
RCT	3 (215)	0.43 (0.20, 0.92)	Random effects	56.0	0.103
Prospective	1 (37)	0.28 (0.12, 0.68)	—	—	—
GV type					
Overall	2 (157)	0.32 (0.19, 0.55)	Fixed effect	7.1	0.300
GOV1+2	2 (95)	**0.52 (0.14, 1.99)**	Random effects	70.0	0.068
Proportion of HCC					
Higher (>median)	2 (157)	0.32 (0.19, 0.55)	Fixed effect	7.1	0.300
Lower (<median)	1 (37)	0.28 (0.12, 0.68)	—	—	—

Variceal obliteration					
GV type					
Overall	2 (157)	1.02 (0.54, 1.95)	Fixed effect	0.0	0.530
GOV1+2	1 (58)	0.91 (0.29, 2.88)	—	—	—
GOV1	1 (150)	0.19 (0.02, 1.66)	—	—	—
Proportion of HCC					
Higher (>median)	2 (157)	1.02 (0.54, 1.95)	Fixed effect	0.0	0.530
Lower (<median)	1 (150)	0.19 (0.02, 1.66)	—	—	—

Treatment sessions					
Study design					
RCT	3 (307)	−0.46 (−1.58, 0.66)	Random effects	95.3	0.000
Prospective	1 (37)	−0.86 (−1.53, −0.18)	—	—	—
GV type					
Overall	2 (157)	0.07 (−0.63, 0.77)	Random effects	78.5	0.031
GOV1+2	1 (37)	−0.86 (−1.53, −0.18)	—	—	—
GOV1	1 (150)	−1.51 (−1.87, −1.15)	—	—	—
Proportion of HCC					
Higher (>median)	2 (157)	0.05 (−0.64, 0.73)	Random effects	78.4	0.032
Lower (<median)	2 (187)	**−0.99 (−1.20, −0.79)**	Fixed effect	0.0	0.852

Complications (overall)^1^					
Study design					
RCT	3 (215)	0.58 (0.32, 1.06)	Fixed effect	0.0	0.386
Prospective	1 (37)	0.93 (0.22, 3.96)	—	—	—
Retrospective	2 (246)	0.95 (0.51, 1.79)	Fixed effect	0.0	0.804
GV type					
Overall	2 (157)	0.68 (0.32, 1.41)	Fixed effect	28.6	0.263
GOV1+2	2 (95)	0.56 (0.24, 1.31)	Fixed effect	0.0	0.393
GOV1	2 (246)	0.95 (0.51, 1.79)	Fixed effect	0.0	0.804
Proportion of HCC					
Higher (>median)	3 (241)	0.70 (0.37, 1.35)	Fixed effect	0.0	0.485
Lower (<median)	2 (199)	0.98 (0.52, 1.84)	Fixed effect	0.0	0.938

Complications (ulcers/ulcer bleeding)					
Study design					
RCT	2 (210)	0.92 (0.03, 33.71)	Random effects	77.9	0.033
Prospective	1 (37)	0.17 (0.01, 3.78)	—	—	—
Retrospective	2 (246)	0.30 (0.14, 0.65)	Fixed effect	0.0	0.994
GV type					
Overall	1 (60)	0.18 (0.03, 0.94)	—	—	—
GOV1+2	1 (37)	0.17 (0.01, 3.78)	—	—	—
GOV1	3 (396)	**0.37 (0.18, 0.76)**	Fixed effect	51.2	0.129
Proportion of HCC					
Higher (>median)	2 (144)	0.21 (0.05, 0.86)	Fixed effect	0.0	0.759
Lower (<median)	3 (349)	0.36 (0.17, 0.74)	Fixed effect	53.6	0.116

Complications (infections)					
Study design					
RCT	4 (365)	1.11 (0.56, 2.18)	Fixed effect	15.4	0.315
Prospective	1 (37)	1.33 (0.25, 7.01)	—	—	—
Retrospective	2 (246)	0.72 (0.35, 1.49)	Fixed effect	0.0	0.742
GV type					
Overall	2 (157)	0.88 (0.38, 2.03)	Fixed effect	0.0	0.941
GOV1+2	2 (95)	1.07 (0.37, 3.06)	Fixed effect	0.0	0.735
GOV1	3 (396)	0.92 (0.46, 1.83)	Fixed effect	54.1	0.113
Proportion of HCC					
Higher (>median)	3 (241)	0.89 (0.42, 1.88)	Fixed effect	0.0	0.995
Lower (<median)	3 (349)	0.98 (0.50, 1.94)	Fixed effect	55.7	0.104

GVO: cyanoacrylate injection; GVL: band ligation; GV: gastric varices; GOV: gastroesophageal varices; HCC: hepatocellular carcinoma; RCT: randomized controlled trial; CI: confidence interval; OR: odds ratio.

^1^Study by El Amin et al. [12] was excluded owing to heterogeneity.

**Table 5:** 

Database	Search strategy
MEDLINE	#1 (Ligation) OR (band* or ligat*)
	#2 (N-butyl-2-cyanoacrylate) OR cyanoacrylate
	#3 ((((Gastric Varices) OR gastric varic*) OR (portal hypertension* or cirrho*)) OR Liver-Cirrhosis) OR hypertension-portal
	#4 #1 AND #2 AND #3

EMBASE	#1 exp LIGATION/
	#2 (band* or ligat*). mp. [mp = title, abstract, subject headings, heading word, drug trade name, original title, device manufacturer, drug manufacturer, device trade name, keyword]
	#3 1 or 2
	#4 exp cyanoacrylate/
	#5 exp N-butyl-2-cyanoacrylate/
	#6 (cyanoacrylate or N-butyl-2-cyanoacrylate). mp. [mp = title, abstract, subject headings, heading word, drug trade name, original title, device manufacturer, drug manufacturer, device trade name, keyword]
	#7 4 or 5 or 6
	#8 exp gastric varices/
	#9 (gastric and varic*). mp. [mp = title, abstract, subject headings, heading word, drug trade name, original title, device manufacturer, drug manufacturer, device trade name, keyword]
	#10 exp portal-hypertension/
	#11 exp liver-cirrhosis/
	#12 (portal hypertension* or cirrho*). mp. [mp = title, abstract, subject headings, heading word, drug trade name, original title, device manufacturer, drug manufacturer, device trade name, keyword]
	#13 8 or 9 or 10 or 11 or 12
	#14 3 and 7 and 13

Cochrane Library	#1 MeSH descriptor Ligation explode all trees
	#2 band* or ligat*
	#3 MeSH descriptor Cyanoacrylate explode all trees
	#4 cyanoacrylate or N-butyl-2-cyanoacrylate
	#5 MeSH descriptor Gastric Varices explode all trees
	#6 gastric varic*
	#7 MeSH descriptor Hypertension, Portal explode all trees
	#8 MeSH descriptor Liver Cirrhosis explode all trees
	#9 portal hypertension* or cirrho*
	#10 (#1 or #2)
	#11 (#3 or #4)
	#12 (#5 or #6 or #7 or #8 or #9)
	#13 (#10 and #11 and #12)

Google Scholar	“cyanoacrylate” AND “band ligation” AND “gastric varices” AND “cirrhosis”
